# Editorial: Better and comparable data on population health in Europe

**DOI:** 10.25646/6223

**Published:** 2019-12-11

**Authors:** Birte Hintzpeter, Anke-Christine Saß, Thomas Ziese

**Affiliations:** Robert Koch Institute, Berlin, Department of Epidemiology and Health Monitoring

**Keywords:** DATA ON POPULATION HEALTH, EHIS 2, EUROPEAN COMPARISON

The European Union (EU) was established with great purpose as a project of peace and to strengthen democratisation and the economy. The institution was founded in 1993 by twelve Member States and has grown steadily since its inception. During this period, the health of the EU population has also improved significantly: life expectancy has increased, living conditions have improved, and progress has been made in health behaviour and provision of health care. Nevertheless, the growing burden associated with noncommunicable diseases, demographic change and health-related inequalities poses challenges to public health and national healthcare systems across Europe. In order to respond to these challenges adequately, reliable representative data are required on the living conditions, health status, health behaviour and the use of health services within each of the countries making up the EU.

Towards the end of the 1990s, the EU decided to establish a set of harmonised health indicators for a common European health information and knowledge system. The ECHI shortlist (European Core Health Indicators) was the result of a number of successive projects and consists of 88 indicators. Figures from international data sources are available for 67 of these indicators. The provision of comparable health data enables comparisons with other EU Member States. The comparison of health data is also aimed at encouraging mutual learning.

The European Health Interview Survey (EHIS), which is focused on noncommunicable diseases, is an important data pool for indicators of health status and health behaviour. The first wave of EHIS was carried out voluntarily by EU Member States between 2006 and 2009. The second wave was prepared as part of a multi-year process of development and discussion. All 28 Member States were legally obliged to conduct the second wave and did so between 2013 and 2015. On the website Statistics Explained, Eurostat provides information on various health topics based on EHIS data, but also on many other topics. In addition to extensive statistical data, the website also provides explanations and background information, as well as analyses of EHIS data in tabular format.

This issue of the Journal of Health Monitoring is based on data from the second wave of EHIS. The articles that follow are also compatible with two previous issues of the Journal of Health Monitoring (1/2017 and 2/2017) in which selected indicators for Germany and the EU were compared. However, the aggregated data on which earlier results were based provide for more restricted analyses. In contrast, the articles in this issues are based on microdata (anonymised original data collected for the EHIS survey from the EU Member States), which were used to evaluate various aspects of health and to spotlight where Germany stands in terms of population health compared with the rest of Europe.

The first article is entitled Partnership, parenthood, employment and self-rated health in Germany and the EU. Most middle-aged women and men adopt one of three central roles in life. This article demonstrates the impact that each of these roles has on self-rated health and reveals the significance of employment within this context. In Germany, no differences were identified for self-rated maternal health by employment status; a finding that also applies to single mothers.

The second article describes Educational differences in the prevalence of behavioural risk factors in Germany and the EU. Educational differences in health behaviour contribute significantly to the development of educational differences in mortality. In Germany and in most other EU Member States, behavioural risk factors are more prevalent among people in lower education groups than among those in higher education groups. This article describes the extent of these educational differences in relation to five risk factors. Overall, the figures place Germany in the mid-range compared with rest of the EU.

In addition to inequalities in health, demographic change is one of the challenges currently facing the EU. Gaertner et al. analyse limitations in activities of daily living in old age. These limitations are studied with the help of two instruments: ADL (activities of daily living) and iADL (instrumental activities of daily living). People in Germany report fewer limitations in their activities of daily living than the EU average. This applies to ADL limitations (for example in walking, eating and using the toilet) and to iADL limitations (such as in shopping, banking and housekeeping).

The article Depressive symptoms in a European comparison describes the age-standardized prevalences of depressive symptoms over the last two weeks. It relies on self-reported data collected from EHIS participants using a country-specific version of the Patient Health Questionnaire (PHQ-8). The prevalence of depressive symptoms in Germany is higher than the EU average. However, when severity is taken into account, the differences between Germany and the EU average only hold true for mild depressive symptoms. In Germany, a depressive symptomology among younger people is identified more frequently than the EU average, while older people reporting depressive symptoms less frequently than the EU average.

The final article European Health Interview Survey (EHIS) 2 – Background and study methodology describes the methodology implemented in EHIS. The article shows that data collection for EHIS is harmonised between EU Member States and, thus, the data collected for the study demonstrates a high degree of comparability. Nevertheless, this contribution also notes the importance of accounting for country-specific issues such as socioeconomic and cultural factors when interpreting the results. As such, it is quite possible that cultural differences between EU Member States play a role when people rate their health or assess limitations and illnesses.

Eurostat publishes a range of health-related data such as national Causes of death statistics from EU Member States on its website. In addition to absolute figures, it also details raw and standardised mortality rates. Furthermore, in addition to EHIS data, Eurostat also publishes data from the EU statistic on income and living conditions (EU-SILC).

EU-wide comparable data on health can contribute towards the development of national strategies aimed at addressing health challenges that also exist in other European countries. Moreover, these data can also be used to identify best practices for policy measures in various countries. However, this relies on a sustainable flow of information and the continual development of health data in the EU.

As the EHIS survey is mandatory throughout the EU, it constitutes an important milestone in improving the health-related information available at the EU level. However, the EU still lacks a sustainable structure or institution with the competence for noncommunicable diseases and their determinants, such as the European Centre for Disease Prevention and Control (EDCD) has in the case of infectious diseases. Many of the monitoring and indicator systems for noncommunicable diseases described above have been implemented during temporary projects. Although such projects generate targeted approaches, they provide no basis with which to deliver sustainable health information and expertise. As such, a sustainable health information system is required that can bring together the EU’s health information. Ideally, this would include the provision of health-related information at the data level, at the informational level in terms of indicators, and at the level of knowledge with regard to the provision of summary reports and assessments. This would improve the basis for developing appropriate and evidence-based public health measures aimed at improving the health of the EU population.

